# Ceftobiprole activity against multidrug-resistant *Staphylococcus aureus* clinical isolates collected in the United States from 2016 through 2022

**DOI:** 10.1128/aac.01402-24

**Published:** 2025-01-13

**Authors:** Helio S. Sader, Jennifer I. Smart, Rodrigo E. Mendes, Mariana Castanheira

**Affiliations:** 1 JMI Laboratories, Element Materials Technology 138461 , North Liberty, Iowa, USA; 2 Basilea Pharmaceutica International Ltd 449688 , Allschwil, Switzerland; The Peter Doherty Institute for Infection and Immunity, Melbourne, Australia

**Keywords:** cephalosporin, ceftobiprole, ceftaroline, MRSA, skin and skin structure infection, pneumonia, bacteremia

## Abstract

Ceftobiprole was recently approved by the United States (US) Food and Drug Administration (FDA) for the treatment of adult patients with *Staphylococcus aureus* bacteremia, including right-side endocarditis, acute bacterial skin and skin structure infections, and community-acquired bacterial pneumonia in adults and pediatrics. Ceftobiprole is an advanced-generation cephalosporin approved in many countries for the treatment of adults with community-acquired pneumonia and hospital-acquired pneumonia, excluding ventilator-associated pneumonia. We evaluated the activities of ceftobiprole and comparators against methicillin-resistant *S. aureus* (MRSA) and multidrug-resistant (MDR) *S. aureus* clinical isolates. A total of 19,764 *S*. *aureus* isolates were collected from patients with various infection types at 37 US medical centers from 2016 to 2022. Susceptibility testing was performed by broth microdilution according to Clinical and Laboratory Standard Institutes (CLSI) standards. Isolates were categorized as MDR if they were nonsusceptible by CLSI criteria to ≥3 antimicrobials. Ceftobiprole was highly active against MRSA (*n* = 8,184; MIC_50/90_, 1/2 mg/L; 99.3% susceptible [S]) and MDR (*n* = 2,789; MIC_50/90_, 1/2 mg/L; 98.1%S) isolates and retained activity against 87.3% of ceftaroline-nonsusceptible isolates (*n* = 433; MIC_50/90_, 2/4 mg/L). Ceftobiprole demonstrated greater susceptibility rates than ceftaroline against all resistant subsets. Ceftobiprole was highly active against isolates nonsusceptible to clindamycin (98.0%S), daptomycin (100.0%S), doxycycline (98.2%S), erythromycin (99.5%S), gentamicin (98.1%S), levofloxacin (99.1%S), tetracycline (99.1%S), tigecycline (100.0%S), and trimethoprim-sulfamethoxazole (99.4%S) and isolates with decreased susceptibility to vancomycin (98.3%S).

## INTRODUCTION

*Staphylococcus aureus* continues to represent a significant cause of infection worldwide, with elevated morbidity and mortality rates. *S. aureus* is a leading cause of both community-acquired and healthcare-associated infections, including bloodstream infections, endocarditis, catheter-related infections, acute bacterial skin and skin structure infections (ABSSSIs), pneumonia, prosthetic joint infections, and osteomyelitis (1). The prevalence of nosocomial infections caused by multidrug-resistant (MDR) *S. aureus* has been markedly elevated in the United States (US) for many years (2–4). Moreover, timely introduction of effective antimicrobial therapy plays an important role in the management of *S. aureus* infections (5).

Ceftobiprole medocaril is a parenteral prodrug of the advanced-generation cephalosporin ceftobiprole and is approved in many European and non-European countries for the treatment of adults with community-acquired bacterial pneumonia (CABP) and hospital-acquired pneumonia (HAP), excluding ventilator-associated pneumonia (VAP) (6). Ceftobiprole was designated by the US Food and Drug Administration (FDA) as a qualified infectious disease product and approved in 2024 by the US FDA for the treatment in adults of *S. aureus* bacteremia, including right-side endocarditis, ABSSSI, and CABP in adults and pediatrics.

Ceftobiprole has a strong affinity for penicillin-binding proteins (PBPs), including PBP2A, which mediates resistance to β-lactams in methicillin-resistant *S. aureus* (MRSA). Ceftobiprole displays potent *in vitro* antimicrobial activity against most clinically important Gram-positive pathogens, and it also exhibits antimicrobial activity against Enterobacterales that is similar to other advanced cephalosporins like cefepime (7). In this study, the activity of ceftobiprole and comparators was evaluated against recent MRSA and MDR *S. aureus* clinical isolates collected in US medical centers.

### Surveillance network and organisms

A total of 19,764 *S*. *aureus* isolates were collected from patients with various infection types at 37 US medical centers from January 2016 to December 2022. These isolates were consecutively collected from patients with bloodstream infection (*n* = 4,766; 24.1%), ABSSSI (*n* = 9,113; 46.1%), pneumonia (*n* = 4,971; 25.2%), intra-abdominal infection (*n* = 690; 3.5%), and other infections (*n* = 224; 1.1%). Each cultured organism was unique (one isolate per patient per infectious episode). All testing was performed in a central reference laboratory (JMI Laboratories/Element Materials Technology, North Liberty, Iowa, USA) under Good Laboratory Practices and Clinical Laboratory Improvement Amendments-certified conditions.

### Antimicrobial susceptibility testing

All organisms were tested for antimicrobial susceptibility by the Clinical and Laboratory Standard Institutes (CLSI) broth microdilution method (8). Comparator agents included those representing the most common classes of drugs used for treatment of *S. aureus* infections. Isolates were categorized as MDR if they were nonsusceptible (CLSI criteria) to ≥3 of the following antimicrobials: clindamycin, daptomycin, erythromycin, gentamicin, levofloxacin, linezolid, tetracycline, tigecycline, trimethoprim-sulfamethoxazole, and vancomycin. Isolates displaying oxacillin MIC values ≥ 4 mg/L were categorized as MRSA. CLSI (9), US FDA (10), and the European Committee on Antimicrobial Susceptibility Testing (EUCAST) (11) interpretive criteria were applied according to current guidelines. For ceftobiprole, the susceptibility breakpoint criteria published by the US FDA (≤2 mg/L for susceptible, 4 mg/L for intermediate, and ≥8 mg/L for resistant) (10) and EUCAST (≤2 mg/L for susceptible and >2 mg/L for resistant) (11) were applied. Quality control strains were also tested to ensure that proper test conditions and procedures were applied. The American Type Culture Collection (ATCC) strains *S. aureus* 29213, *Enterococcus faecalis* 29212, and *Escherichia coli* 25922 were tested on every run. Other ATCC strains tested during the study include *S. aureus* 43300 (MRSA), *Streptococcus pneumoniae* 49619, and *Pseudomonas aeruginosa* 27853. The inoculum density during susceptibility testing was monitored by bacterial colony counts.

Overall, 99.7% of *S. aureus* isolates were susceptible (MIC, ≤2 mg/L) to ceftobiprole (MIC_50/90_, 0.5/2 mg/L; Table 1). The highest ceftobiprole MIC value among MSSA isolates was 1 mg/L (MIC_50/90_, 0.5/0.5 mg/L; Table 1). Ceftobiprole remained highly active against MDR isolates (*n* = 2,789), inhibiting 98.1% of isolates at the US FDA and EUCAST susceptible breakpoint of ≤2 mg/L (MIC_50/90_, 1/2 mg/L). Fifty-three (1.9%) MDR isolates had ceftobiprole MIC ≥4 mg/L; 50 isolates at 4 mg/l (1.8%), which is classified as intermediate by the US FDA breakpoint criteria; and only three isolates (0.1%) at >4 mg/L. Ceftobiprole exhibited potent activity against the MSSA MDR isolates (MIC_50/90_, 0.5/0.5 mg/L; 100.0% susceptible [S]) and MRSA MDR isolates (MIC_50/90_, 1/2 mg/L; 97.8%S; Table 1). The overall MRSA rate was 41.4%, and 99.3% of MRSA isolates were ceftobiprole susceptible (MIC_50/90_, 1/2 mg/L; Tables 1 and 2).

**TABLE 1:** Cumulative distributions of MIC values for ceftobiprole against the various *S. aureus*-resistant subsets^*b*^

Subset (no. of isolates)	No. and cumulative % of isolates inhibited at MIC (mg/L) of:									MIC_50_	MIC_90_	%S^*a*^
	≤0.03	0.06	0.12	0.25	0.5	1	2	4	>4			
*S. aureus* (all; 19,764)	5 <0.1	13 0.1	96 0.6	3,683 19.2	8,267 61.0	5,723 90.0	1,918 99.7	56 >99.9	3 100.0	0.5	2	99.7
MSSA (11,580)	5 <0.1	13 0.2	92 0.9	3,659 32.5	7,786 99.8	25 100.0				0.5	0.5	100.0
MRSA (8,184)		0 0.0	4 <0.1	24 0.3	481 6.2	5,698 75.8	1,918 99.3	56 >99.9	3 100.0	1	2	99.3
Ceftobiprole-NS (>2 mg/L) (59)							0 0.0	56 94.9	3 100.0	4	4	0.0
Ceftaroline-NS (>1 mg/L) (433)					0 0.0	5 1.2	373 87.3	52 99.3	3 100.0	2	4	87.3
Clindamycin-NS (>0.5 mg/L) (2,618)		0 0.0	11 0.4	178 7.2	382 21.8	941 57.8	1,053 98.0	50 99.9	3 100.0	1	2	98.0
Daptomycin-NS (>1 mg/L) (8)			0 0.0	1 12.5	4 62.5	2 87.5	1 100.0			0.5		100.0
Doxycycline-NS (>4 mg/L) (393)		0 0.0	2 0.5	40 10.7	112 39.2	119 69.5	113 98.2	6 99.7	1 100.0	1	2	98.2
Erythromycin-NS (>0.5 mg/L) (11,026)	0 0.0	3 <0.1	28 0.3	1,016 9.4	3,300 39.4	4,795 82.9	1,826 99.5	56 >99.9	2 100.0	1	2	99.5
Gentamicin-NS (>4 mg/L) (419)			0 0.0	30 7.2	133 38.9	132 70.4	116 98.1	8 100.0		1	2	98.1
Levofloxacin-NS (>1 mg/L) (6,477)	0 0.0	2 <0.1	12 0.2	330 5.3	1,034 21.3	3,366 73.2	1,674 99.1	56 >99.9	3 100.0	1	2	99.1
Teicoplanin MIC of 4 mg/L (12)				0 0.0	6 50.0	1 58.3	5 100.0			0.5	2	100.0
Tetracycline-NS (>4 mg/L) (1,184)	0 0.0	1 0.1	9 0.8	121 11.1	419 46.5	426 82.4	197 99.1	8 99.7	3 100.0	1	2	99.1
Tigecycline-NS (>0.5 mg/L) (5)			0 0.0	2 40.0	0 40.0	0 40.0	3 100.0			2		100.0
TMP-SMX-NS (>2 mg/L) (490)	0 0.0	1 0.2	0 0.2	15 3.3	84 20.4	313 84.3	74 99.4	0 99.4	3 100.0	1	2	99.4
Vancomycin MIC of 2 mg/L (117)			0 0.0	24 20.5	39 53.8	29 78.6	23 98.3	2 100.0		0.5	2	98.3
MDR *S. aureus* (2,789)		0 0.0	7 0.3	135 5.1	338 17.2	1,107 56.9	1,149 98.1	50 99.9	3 100.0	1	2	98.1
MDR MRSA (2,421)		0 0.0	1 <0.1	8 0.4	104 4.7	1,106 50.4	1,149 97.8	50 99.9	3 100.0	1	2	97.8
MDR MSSA (368)		0 0.0	6 1.6	127 36.1	234 99.7	1 100.0				0.5	0.5	100.0

^*a*^ Percentage susceptible per US FDA and EUCAST criteria (≤2 mg/L).

^*b*^ MSSA, methicillin-susceptible *S. aureus*; MRSA, methicillin-resistant *S. aureus*; NS, nonsusceptible per CLSI criteria; TMP-SMX, trimethoprim-sulfamethoxazole; MDR, multidrug-resistant.

**TABLE 2:** Activity of ceftobiprole and comparator agents against *S. aureus*

Antimicrobial agent	MIC in mg/L		CLSI^*a*^			EUCAST^*b*^		
	MIC_50_	MIC_90_	%S	%I	%R	%S	%I	%R
MDR (2,789)								
Ceftobiprole	1	2	98.1	1.8	0.1	98.1		1.9
Ceftaroline	1	2	86.9	13.0^*c*^	<0.1	86.9^*d*^ 86.9^*e*^	12.9^*d*^	0.1^*d*^ 13.1^*e*^
Clindamycin	>2	>2	17.3	1.1	81.6	17.2		82.8
Daptomycin	0.25	0.5	99.9			99.9		0.1
Doxycycline	0.12	8	89.0	9.7	1.3	80.8		19.2
Erythromycin	>8	>8	0.6	1.3	98.0	0.8		99.2
Gentamicin	≤1	2	90.1	0.3	9.6	90.1^*f*^		9.9
Levofloxacin	>4	>4	4.6	0.7	94.7	^*g*^	4.6	95.4
Linezolid	1	2	>99.9		<0.1	>99.9		<0.1
Minocycline	0.12	4	90.5	3.8	5.8	85.2		14.8
Oxacillin	>2	>2	13.2		86.8	13.2		86.8
Teicoplanin	≤0.5	≤0.5	100.0	0.0	0.0	99.8		0.2
Tetracycline	≤0.5	>8	76.6	1.3	22.1	72.6		27.4
Tigecycline	0.12	0.25	99.9^*h*^			99.9		0.1
TMP-SMX^*i*^	≤0.5	>4	85.6		14.4	85.6	0.7	13.7
Vancomycin	1	1	100.0	0.0	0.0	100.0		0.0
MRSA (8,184)								
Ceftobiprole	1	2	99.3	0.7	<0.1	99.3		0.7
Ceftaroline	0.5	1	94.7^*c*^	5.3	<0.1	94.7^*d*^ 94.7^*e*^	5.2^*d*^	<0.1^*d*^ 5.3^*e*^
Clindamycin	≤0.25	>2	73.9	0.4	25.7	73.8		26.2
Daptomycin	0.25	0.5	>99.9			>99.9		<0.1
Doxycycline	≤0.06	1	96.7	3.0	0.3	93.8		6.2
Erythromycin	>8	>8	14.0	3.1	82.9	14.3		85.7
Gentamicin	≤1	≤1	96.5	0.2	3.3	96.5^*f*^		3.5
Levofloxacin	4	>4	34.2	0.8	65.0	^*g*^	34.2	65.8
Linezolid	1	2	>99.9		<0.1	>99.9		<0.1
Minocycline	≤0.06	0.25	97.4	1.2	1.5	95.4		4.6
Oxacillin	>2	>2	0.0		100.0	0.0		100.0
Teicoplanin	≤0.5	≤0.5	100.0	0.0	0.0	99.9		0.1
Tetracycline	≤0.5	2	91.4	1.2	7.4	89.1		10.9
Tigecycline	0.06	0.12	>99.9^*h*^			>99.9		<0.1
TMP-SMX^*i*^	≤0.5	≤0.5	94.9		5.1	94.9	0.2	4.9
Vancomycin	1	1	100.0	0.0	0.0	100.0		0.0
MDR MRSA (2,421)								
Ceftobiprole	1	2	97.8	2.1	0.1	97.8		2.2
Ceftaroline	1	2	85.0^*c*^	15.0	<0.1	85.0^*d*^ 85.0^*e*^	14.9^*d*^	0.2^*d*^ 15.0^*e*^
Clindamycin	>2	>2	17.4	1.0	81.6	17.3		82.7
Daptomycin	0.25	0.5	99.9			99.9		0.1
Doxycycline	0.12	4	90.1	8.8	1.0	82.4		17.6
Erythromycin	>8	>8	0.5	1.1	98.3	0.6		99.4
Gentamicin	≤1	≤1	90.3	0.3	9.4	90.3^*f*^		9.7
Levofloxacin	>4	>4	1.8	0.7	97.4	^*g*^	1.8	98.2
Linezolid	1	2	>99.9		<0.1	>99.9		<0.1
Minocycline	0.12	4	91.7	3.6	4.8	86.6		13.4
Oxacillin	>2	>2	0.0		100.0	0.0		100.0
Teicoplanin	≤0.5	≤0.5	100.0	0.0	0.0	99.8		0.2
Tetracycline	≤0.5	>8	78.6	1.0	20.4	74.2		25.8
Tigecycline	0.12	0.25	99.8^*h*^			99.8		0.2
TMP-SMX^*i*^	≤0.5	>4	84.7		15.3	84.7	0.7	14.5
Vancomycin	1	1	100.0	0.0	0.0	100.0		0.0
MDR MSSA (368)								
Ceftobiprole	0.5	0.5	100.0	0.0	0.0	100.0		0.0
Ceftaroline	0.25	0.25	100.0^*c*^	0.0	0.0	100.0^*d*^ 100.0^*e*^	0.0^*d*^	0.0^*d*^ 0.0^*e*^
Clindamycin	>2	>2	16.6	1.4	82.1	16.6		83.4
Daptomycin	0.25	0.5	99.7			99.7		0.3
Doxycycline	0.12	8	81.8	15.2	3.0	70.1		29.9
Erythromycin	>8	>8	1.4	2.7	95.9	1.6		98.4
Gentamicin	≤1	>8	88.6	0.0	11.4	88.6*f*		11.4
Levofloxacin	>4	>4	22.6	0.5	76.9	^*g*^	22.6	77.4
Linezolid	1	2	100.0		0.0	100.0		0.0
Minocycline	0.12	>8	82.6	4.9	12.5	75.8		24.2
Oxacillin	0.5	1	100.0		0.0	100.0		0.0
Teicoplanin	≤0.5	≤0.5	100.0^*h*^	0.0	0.0	99.5		0.5
Tetracycline	≤0.5	>8	63.3	3.3	33.4	61.7		38.3
Tigecycline	0.12	0.25	100.0			100.0		0.0
TMP-SMX^*i*^	≤0.5	1	91.3		8.7	91.3	0.5	8.2
Vancomycin	1	1	100.0	0.0	0.0	100.0		0.0

^*a*^ Criteria as published by CLSI and/or US FDA US FDA (2024).

^*b*^ Criteria as published by EUCAST (2024).

^*c*^ Intermediate is interpreted as susceptible–dose-dependent.

^*d*^ Using other than pneumonia breakpoints.

^*e*^ Using pneumonia breakpoints.

^*f*^ For systemic infections, aminoglycosides must be used in combination with other active therapy.

^*g*^ An arbitrary susceptible breakpoint of ≤0.001 mg/L has been published by EUCAST indicating that susceptible should not be reported for this organism-agent combination and intermediate should be interpreted as susceptible–increased exposure (EUCAST, 2024).

^*h*^ Criteria as published by the US FDA US FDA (2024).

^*i*^ MDR, multidrug-resistant; MRSA, methicillin-resistant *S. aureus*; TMP-SMX, trimethoprim-sulfamethoxazole.

Notably, ceftobiprole retained activity against isolates resistant to antimicrobial agents used to treat *S. aureus* infections, including the anti-MRSA agent ceftaroline. Among 433 ceftaroline-nonsusceptible isolates (MIC > 1 mg/L), 378 (87.3%) were susceptible (MIC_50/90_, 2/4 mg/L), 50 (12.0%) were intermediate (MIC 4 mg/L), and 3 (0.7%) were resistant (MIC > 4 mg/L) to ceftobiprole (Table 1). Moreover, ceftobiprole was active against isolates nonsusceptible to clindamycin (98.0%S), daptomycin (100.0%S), doxycycline (98.2%S), erythromycin (99.5%S), gentamicin (98.1%S), levofloxacin (99.1%S), tetracycline (99.1%S), tigecycline (100.0%S), and trimethoprim-sulfamethoxazole (99.4%S), as well as against isolates with decreased susceptibility to teicoplanin (100.0%S) or vancomycin (98.3%S; Table 1).

The activity of ceftobiprole and comparator agents against 2,789 MDR *S. aureus* and 8,184 MRSA isolates are shown in Table 2. Susceptibility rates were higher for ceftobiprole (98.1%) than ceftaroline (86.9%) (10, 11). The most active compounds tested against MDR isolates were vancomycin (100.0%S), teicoplanin (100.0%S/99.8%S per CLSI/EUCAST), linezolid (>99.9%S), daptomycin (99.9%S), tigecycline (99.9%S), and ceftobiprole (98.1%S; Table 2). Among MRSA isolates, 99.3% were susceptible to ceftobiprole, and 94.7% were susceptible to ceftaroline (Table 2).

Comparison of ceftobiprole and ceftaroline activity against various resistant subsets is summarized in Table 3. Ceftobiprole demonstrated greater susceptibility rates against all resistant subsets except the ceftobiprole-nonsusceptible subset. Highest differences in susceptibility rates were observed for MDR MRSA (12.8%), gentamicin-nonsusceptible (11.0%), clindamycin-nonsusceptible (11.7%), MDR (11.2%), levofloxacin-nonsusceptible (5.7%), and isolates with decreased susceptibility to vancomycin (5.2%; Table 3).

**TABLE 3:** Comparison activity of ceftobiprole and ceftaroline against various *S. aureus*-resistant subsets^*c*^

Group	Number of isolates	% Susceptible	
		Ceftobiprole^*a*^	Ceftaroline^*b*^
All	19,764	99.7	97.8
MDR	2,789	98.1	86.9
MRSA	8,184	99.3	94.7
MDR MRSA	2,421	97.8	85.0
MDR MSSA	368	100.0	100.0
Ceftaroline-NS (>1 mg/L)^*b*^	433	87.3	0.0
Ceftobiprole-I (4 mg/L)^*a*^	59	0.0	6.8
Clindamycin-NS (>0.5 mg/L)	2,618	98.0	86.3
Doxycycline-NS (>4 mg/L)	393	98.2	88.0
Erythromycin-NS (>0.5 mg/L)	10,940	99.5	96.1
Gentamicin-NS (>4 mg/L)	419	98.1	87.1
Levofloxacin-NS (>1 mg/L)	6,477	99.1	93.4
Tetracycline-NS (>4 mg/L)	1,184	99.1	94.6
TMP-SMX-NS (>2 mg/L)	490	99.4	98.4
Vancomycin MIC of 2 mg/L	117	98.3	93.1

^*a*^ Applying susceptible breakpoint of ≤2 mg/L (US FDA and EUCAST).

^*b*^ Applying susceptible breakpoint of ≤1 mg/L (US FDA, CLSI, and EUCAST).

^*c*^ MDR, multidrug-resistant; MRSA, methicillin-resistant *S. aureus*; NS, nonsusceptible, I, intermediate; TMP-SMX, trimethoprim-sulfamethoxazole.

The cephalosporin class has evolved greatly through the development of new-generation molecules that are active against MRSA and retain a wide spectrum of activity against Gram-positive and Gram-negative pathogens (6). Previous studies from our group and other investigators have shown that ceftobiprole is highly active against staphylococci, including MRSA, and streptococcal species, including penicillin- and ceftriaxone-resistant *S. pneumoniae* (7, 12, 13). Ceftobiprole has also demonstrated activity against Enterobacterales (7, 12–14).

The objective of this investigation was focused on the evaluation of ceftobiprole activity against various subsets of MDR *S. aureus* isolates from patients hospitalized in US medical centers in a 7-year period (2016–2022). First, our findings confirm previously published results on the activity of ceftobiprole against MRSA by showing that only 59 of 8,184 isolates tested (0.7%) had an MIC value greater than the US FDA and EUCAST susceptible breakpoint of ≤2 mg/L; 56 of these isolates had a ceftobiprole MIC of 4 mg/L, which is categorized as intermediate (Table 1). Pfaller et al. (7) evaluated 558 MRSA isolates collected from US hospitals in 2016 and 2017. The study showed that 99.3% of isolates were inhibited at ≤2 mg/L of ceftobiprole and the remaining 0.7% had a ceftobiprole MIC of 4 mg/L. More recently, Canton et al. (12) evaluated a large collection of Gram-positive and Gram-negative bacteria, including 3,502 MRSA isolates and found that 99.5% of MRSA were susceptible to ceftobiprole. The remaining 0.5% (19 isolates) had ceftobiprole MIC of 4 mg/L.

Furthermore, the results of this study expand the data published by other investigators by evaluating the ceftobiprole activity against a large collection of MDR *S. aureus* isolates. Among 2,789 MDR isolates tested, 2,421 (86.8%) were MRSA. Ceftobiprole was active against 98.1% of all MDR isolates (MIC_50/90_, 1/2 mg/L) and 97.8% of MDR MRSA isolates (MIC_50/90_, 1/2 mg/L). MDR isolates that were susceptible to oxacillin (MSSA) were highly susceptible to ceftobiprole (MIC_50/90_, 0.5/0.5 mg/L; 100.0%S). Our results also indicated that ceftobiprole activity does not appear to be affected by mechanisms that cause resistance or decreased susceptibility to antimicrobial agents commonly used to treat MSSA or MRSA infections, including vancomycin (98.3% of isolates with vancomycin MIC of 2 mg/L were susceptible to ceftobiprole [2 mg/L was the highest vancomycin MIC value observed, and it is still on the vancomycin susceptible range]), clindamycin, daptomycin (eight daptomycin-nonsusceptible isolates were tested, and all were susceptible to ceftobiprole), doxycycline, erythromycin, gentamicin, levofloxacin, tetracycline, tigecycline, and trimethoprim-sulfamethoxazole (Table 1).

One of the most important findings of this investigation was the fact that ceftobiprole retained activity against 87.3% (378/433) of isolates that were not susceptible to ceftaroline, which is the only other anti-MRSA advanced-generation cephalosporin available for clinical use (15). The activity of both ceftobiprole and ceftaroline against MRSA is attributed to the ability of these compounds to acylate and inhibit the MRSA PBP, PBP2A, while maintaining affinity for the other essential PBPs, such as PBP1, PBP2, and PBP3. Furthermore, resistance or decreased susceptibility to these compounds has been attributed mostly to alterations in the amino acid sequence of PBP2A (16–18).

The US FDA established ceftobiprole breakpoints for *S. aureus* of ≤2 mg/L for susceptible, 4 mg/L for intermediate, and ≥8 for resistant (10) based on clinical outcome data from three Phase 3 studies in SAB, ABSSSI, and CABP and a probability of target attainment of 100% at an MIC of 4 mg/L for *S. aureus*. The ceftobiprole EUCAST *S. aureus* breakpoints are ≤2 mg/L for susceptible and ≥4 mg/L for resistant, with a pharmacokinetic/pharmacodynamic (PK/PD) breakpoint of 4 mg/L based on >90% target attainment at an MIC of 4 mg/L (10, 11). EUCAST clinical breakpoints were set in 2016 based on the CABP and HAP Phase 3 clinical data and PK/PD analyses of these data sets. *S. aureus* isolates with a ceftobiprole MIC > 4 mg/L have not been detected in any of the Phase 3 clinical studies.

The US FDA recently reevaluated the ceftaroline susceptible-dose dependent (SDD) breakpoint originally proposed by CLSI in 2019. CLSI proposed a SDD breakpoint for ceftaroline based on MIC distribution data showing a prevalence of higher ceftaroline MIC values (2 and 4 mg/L) in parts of the world other than the United States. The FDA evaluated the available data for the ceftaroline 600 mg every 8 hours with a 2-hour infusion dosing regimen and did not find any safety concerns though there is limited clinical efficacy data available for ceftaroline against *S. aureus* isolates with MICs > 2 mg/L. Surveillance studies have shown a significant number of *S. aureus* isolates with ceftaroline MICs of 2 mg/L or greater. The new FDA ceftaroline SDD breakpoint is 2–4 mg/L with a resistant breakpoint of 8 mg/L for the new dosing regimen (11).

Ceftobiprole is the only approved β-lactam for the treatment of SAB due to MRSA. Clinical data comparing these two anti-MRSA cephalosporins is scarce. Zampino et al. (19) evaluated 138 patients, 63 treated with ceftobiprole, and 75 treated with ceftaroline in a retrospective study. Ceftobiprole and ceftaroline were applied in different clinical scenarios, and both compounds were largely used in combination with other antibiotics. Patients treated with ceftobiprole had more comorbidities and a higher prevalence of multiple-site infections and were more often treated empirically, whereas ceftaroline was more frequently used in patients with healthcare-associated infections. The investigators concluded that ceftaroline and ceftobiprole were comparable in terms of clinical efficacy and tolerability and found no differences in terms of hospital mortality, length of stay, and rates of clinical cure, improvement, or failure (19).

Garcia et al. (20) compared the real-life effectiveness and safety of ceftobiprole and ceftaroline in a retrospective, observational, multicenter study. The study was performed in Spain and included patients receiving outpatient parenteral antimicrobial therapy and hospitalized patients treated for at least 48 hours. Ceftobiprole was administered to 212 patients and ceftaroline to 227 patients. Ceftobiprole was more frequently administered against polymicrobial infections, Gram-negative infections, nosocomial pneumonia, and ABSSSI. Ceftaroline-treated patients were younger and had higher rates of septic shock, higher rates of infection due to Gram-positive cocci, bacteremia, infective endocarditis, and VAP. Patients receiving ceftaroline had a longer hospital stay with no difference in infection-related mortality at 14 or 28 days or in dropout due to adverse effects. Thus, the authors concluded that both advanced-generation cephalosporins are safe and effective in real life (20).

In summary, ceftobiprole demonstrated potent *in vitro* activity and almost complete coverage against MRSA (99.3%S) and MDR (98.1%S) *S. aureus* from US hospitals. Higher susceptibility rates for ceftobiprole than ceftaroline may suggest consideration for ceftobiprole use in patients with MRSA isolates with decrease susceptibility to ceftaroline. The recently completed ceftobiprole Phase 3 clinical trials have shown that the FDA-approved dose for ceftobiprole has been optimized to cover patients against difficult to treat infections due to MRSA and MDR *S. aureus*. Additional head-to-head studies comparing the clinical efficacy of ceftobiprole and ceftaroline at the increased dosage are warranted to better understand the role of these compounds for the treatment of MRSA infections.

## References

[1] Linz MS, Mattappallil A, Finkel D, Parker D. 2023. Clinical impact of Staphylococcus aureus skin and soft tissue infections. Antibiotics (Basel) 12:557. doi:10.3390/antibiotics12030557 36978425 PMC10044708

[2] Diekema DJ, Pfaller MA, Shortridge D, Zervos M, Jones RN. 2019. Twenty-year trends in antimicrobial susceptibilities among Staphylococcus aureus from the SENTRY Antimicrobial Surveillance Program. Open Forum Infect Dis 6:S47–S53. doi:10.1093/ofid/ofy270 30895214 PMC6419894

[3] Sader HS, Mendes RE, Streit JM, Flamm RK. 2017. Antimicrobial susceptibility trends among Staphylococcus aureus isolates from U.S. hospitals: results from 7 years of the ceftaroline (AWARE) surveillance program, 2010 to 2016. Antimicrob Agents Chemother 61:e01043-17. doi:10.1128/AAC.01043-17 28630196 PMC5571371

[4] Holland TL, Cosgrove SE, Doernberg SB, Jenkins TC, Turner NA, Boucher HW, Pavlov O, Titov I, Kosulnykov S, Atanasov B, Poromanski I, Makhviladze M, Anderzhanova A, Stryjewski ME, Assadi Gehr M, Engelhardt M, Hamed K, Ionescu D, Jones M, Saulay M, Smart J, Seifert H, Fowler VG Jr, ERADICATE Study Group. 2023. Ceftobiprole for treatment of complicated Staphylococcus aureus bacteremia. N Engl J Med 389:1390–1401. doi:10.1056/NEJMoa2300220 37754204

[5] Al Sidairi H, Reid EK, LeBlanc JJ, Sandila N, Head J, Davis I, Bonnar P. 2023. Optimizing treatment of Staphylococcus aureus bloodstream infections following rapid molecular diagnostic testing and an antimicrobial stewardship program intervention. Microbiol Spectr 11:e0164822. doi:10.1128/spectrum.01648-22 36790177 PMC10101007

[6] Lupia T, Pallotto C, Corcione S, Boglione L, De Rosa FG. 2021. Ceftobiprole perspective: current and potential future indications. Antibiotics (Basel) 10:170. doi:10.3390/antibiotics10020170 33567771 PMC7915564

[7] Pfaller MA, Flamm RK, Duncan LR, Shortridge D, Smart JI, Hamed KA, Mendes RE, Sader HS. 2019. Ceftobiprole activity when tested against contemporary bacteria causing bloodstream infections in the United States (2016-2017). Diagn Microbiol Infect Dis 94:304–313. doi:10.1016/j.diagmicrobio.2019.01.015 30808530

[8] CLSI. 2018. M07Ed11. Methods for dilution antimicrobial susceptibility tests for bacteria that grow aerobically. Wayne, PA.

[9] CLSI. 2024. M100Ed34. Performance standards for antimicrobial susceptibility testing: 34th informational supplement. Wayne, PA.

[10] US FDA. 2024. US FDA-recognized antimicrobial susceptibility test interpretive criteria. Washington, D.C. Available from: https://www.fda.gov/drugs/development-resources/antibacterial-susceptibility-test-interpretive-criteria

[11] EUCAST. 2024. Breakpoint tables for interpretation of MIC’s and zone diameters version 14.0. Available from: https://www.eucast.org/fileadmin/src/media/PDFs/EUCAST_files/Breakpoint_tables/v_14.0_Breakpoint_Tables.pdf

[12] Canton R, Hamed K, Wiktorowicz T, Redder N, Jemmely N, Quevedo J, Santerre Henriksen A. 2022. In vitro activity of ceftobiprole and comparator antibiotics against contemporary European isolates (2016-19). JAC Antimicrob Resist 4:dlac030. doi:10.1093/jacamr/dlac030 35350131 PMC8947215

[13] Santerre Henriksen A, Smart JI, Hamed K. 2018. Susceptibility to ceftobiprole of respiratory-tract pathogens collected in the United Kingdom and Ireland during 2014-2015. Infect Drug Resist 11:1309–1320. doi:10.2147/IDR.S176369 30214251 PMC6118268

[14] Pfaller MA, Flamm RK, Mendes RE, Streit JM, Smart JI, Hamed KA, Duncan LR, Sader HS. 2019. Ceftobiprole activity against Gram-positive and -negative pathogens collected from the United States in 2006 and 2016. Antimicrob Agents Chemother 63:e01566-18. doi:10.1128/AAC.01566-18 30373807 PMC6325186

[15] Abate G, Wang G, Frisby J. 2022. Ceftaroline: systematic review of clinical uses and emerging drug resistance. Ann Pharmacother 56:1339–1348. doi:10.1177/10600280221082326 35300514

[16] Lahiri SD, Alm RA. 2016. Identification of non-PBP2a resistance mechanisms in Staphylococcus aureus after serial passage with ceftaroline: involvement of other PBPs. J Antimicrob Chemother 71:3050–3057. doi:10.1093/jac/dkw282 27494915

[17] Lovering AL, Gretes MC, Safadi SS, Danel F, de Castro L, Page MGP, Strynadka NCJ. 2012. Structural insights into the anti-methicillin-resistant Staphylococcus aureus (MRSA) activity of ceftobiprole. J Biol Chem 287:32096–32102. doi:10.1074/jbc.M112.355644 22815485 PMC3442540

[18] Mendes RE, Tsakris A, Sader HS, Jones RN, Biek D, McGhee P, Appelbaum PC, Kosowska-Shick K. 2012. Characterization of methicillin-resistant Staphylococcus aureus displaying increased MICs of ceftaroline. J Antimicrob Chemother 67:1321–1324. doi:10.1093/jac/dks069 22398650

[19] Zampino R, Gallo R, Salemme A, Marrazzo T, Iossa D, Karruli A, Andini R, Esitini D, Moretto SM, De Gregorio F, Durante-Mangoni E. 2023. Clinical results with the use of ceftaroline and ceftobiprole: real-life experience in a tertiary care hospital. Int J Antimicrob Agents 62:106883. doi:10.1016/j.ijantimicag.2023.106883 37302772

[20] Arnés García D, Pitto-Robles I, Calderón Parra J, Calvo Salvador M, Herrero Rodríguez C, Gisbert L, Hidalgo-Tenorio C. 2023. Ceft-to-Ceft Study: real-life experience with ceftaroline and ceftobiprole in treatment of the principal infectious syndromes in a Spanish multicenter hospital cohort. Antibiotics (Basel) 12:1692. doi:10.3390/antibiotics12121692 38136726 PMC10740782

